# A Bio‐Functional Mimetic Robot for Versatile Tasks From Cross‐Scale Manipulation to Limb‐Tool Integration

**DOI:** 10.1002/advs.76527

**Published:** 2026-08-13

**Authors:** Xin Tong, Tianle Zhang, Fei Mo, Qian Zhao, Zhongqing Sun, Yongkang Jiang, Yang Yang, Yingtian Li

**Affiliations:** ^1^ Shenzhen Institutes of Advanced Technology Chinese Academy of Sciences Shenzhen China; ^2^ School of Automation Nanjing University of Information Science and Technology Nanjing China; ^3^ State Key Laboratory of Autonomous Intelligent Unmanned Systems Tongji University Shanghai China

**Keywords:** bio‐functional mimetic robot, cross‐scale manipulation, self‐growing upper limb, tool‐hand integration

## Abstract

Dexterous hands and robotic manipulators are essential physical interfaces for interacting with diverse environments. Traditional methods seek creature‐like dexterity through structural biomimicry of biological systems, a strategy that results in increased mechanical complexity and control challenges due to the stacking of components. This study presents the BioflexBot, a functionally biomimetic robot that moves beyond structural replication. Utilizing dual air chambers and flexible coiled springs, it achieves three fundamental motions: extension, expansion, and contraction, controlled by only two inputs. These core movements, enabled by an optimized structural design, unlock three distinct functional domains, i.e., human‐like grasping through bio‐functional mimicry, adaptive growth surpassing human biological limits (reaching a 7:1 extension‐to‐contraction ratio), and enhanced performance via tool‐hand integration. Experimental validation demonstrates that BioflexBot can manipulate objects up to 12.9 times larger than comparable systems, leveraging its lightweight, expandable design. This robot also supports diverse applications, including safe end‐effectors for humanoid robots and in situ inspection of aeroengine blades within confined spaces. This work establishes that bio‐functional mimetic design empowers simple mechanical structures to deliver robust multifunctionality, offering a novel solution that overcomes the longstanding trade‐off between versatility and complexity in robotic manipulation.

## Graphical abstract

A functionally biomimetic design methodology is presented, enabling a single robot to perform eight distinct functions and manipulate objects across a size range exceeding 2300‐fold. The robot exceeds basic human hand functions and demonstrates remote operation, multi‐object manipulation, humanoid‐robot hand interaction, and narrow‐space inspection, offering a versatile solution that supports further exploration of its capabilities.

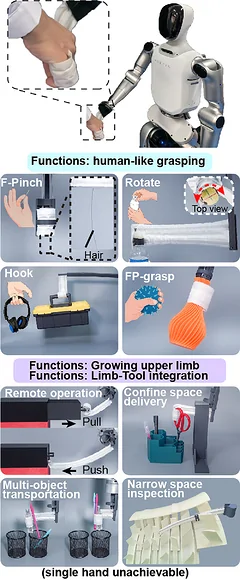

## 1 Introduction

Dexterous robotic hands and manipulators serve as the core physical interface for interacting with the environment, playing a critical role across diverse applications [1, 2, 3]. Traditionally, efforts to achieve life‐like dexterity have centered on structural biomimicry, which directly emulates biological forms like elephant trunks [4], octopus tentacles [5], and human hands [6]. The human hand is renowned for its extraordinary functional versatility, a complexity arising from bones intricately coordinated by an elaborate muscular system [7]. However, replicating this intricacy in mechanical systems through stacking numerous components inevitably leads to increased structural complexity [7, 8, 9] and substantial control challenges [10, 11], highlighting an urgent need for efficient, practical, and robust robot designs.

**FIGURE 1:**
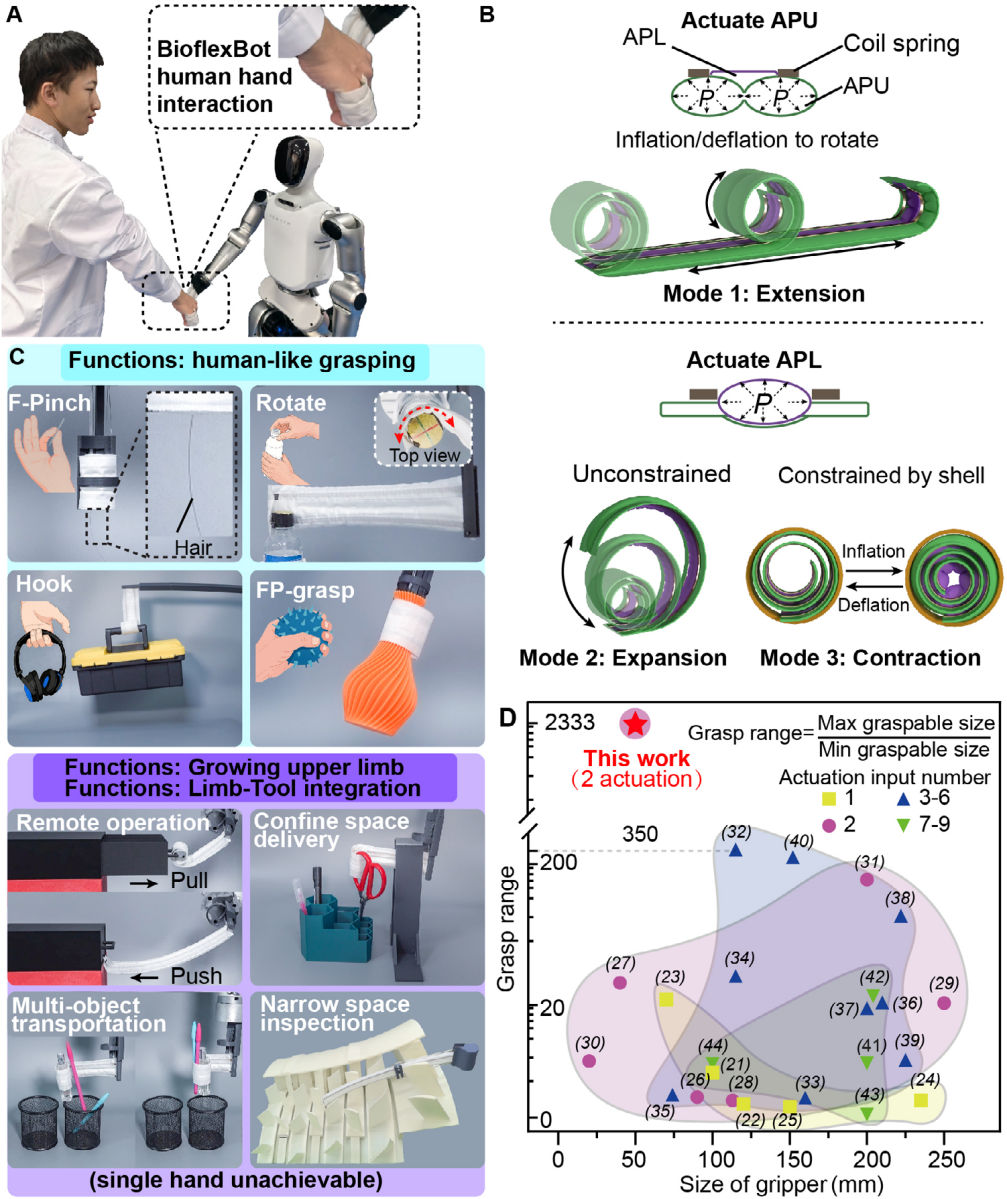
Design and functions of the BioflexBot. (A) BioflexBot serves as a dexterous end‐effector for humanoid robots, enabling safe and intuitive human‐robot interaction. (B) The schematic illustration and cross‐sectional view present the structure of the robot and its actuation processes to realize three fundamental deformation modes of our robot, i.e., extension, expansion, and contraction. (C)Realizing hand function through bio‐functional mimicry of human grasping and performing tasks beyond biological limits via an upper limb with adaptive growing capabilities, and tool‐hand integration. (D) Comparison between the grasp range and physical size of the BioflexBot and that of previously developed grippers, grouped by the number of actuation inputs: single‐actuator Refs. [21, 22, 23, 24, 25], dual‐actuator Refs. [26, 27, 28, 29, 30, 31], 3–6 actuators Refs. [32, 33, 34, 35, 36, 37, 38, 39, 40], and 7–9 actuators Refs. [41, 42, 43, 44].

To overcome this limitation, we propose a fundamental methodology change from mimicking biological structures to abstracting and implementing core biological functions. This approach aims to break the longstanding trade‐off between functional versatility and mechanical complexity that has constrained traditional robotic hand development. Our research, rather than duplicating the hand's complex anatomy, focuses on reproducing and extending its essential functions using structurally simple yet elegant designs. We abstract the human hand's core manipulation capabilities [12], i.e., pinching, rotating, hooking, and enveloping, and aim to surpass its physical limitations through elegant design. Here, we introduce a BioflexBot, a bio‐functional mimetic robot that embodies this paradigm. As illustrated in Figure 1A, the proposed BioflexBot is intended to partially substitute current humanoid dexterous hands, facilitating safer human‐robot interaction. Fabricated via a multi‐layer heating press process (Figure S1), its body performs three core motions, i.e., extension, expansion, and contraction, controlled by only two pneumatic inputs, as shown in Figure 1B. By encoding functionality directly into its morphing structure, the BioflexBot achieves three versatile functions: 1) bio‐functional mimicry of human grasping, 2) an upper limb with adaptive growing ability, and 3) tool‐hand integration. The first function replicates the four fundamental manipulation modes of human hand (top of Figure 1C). The latter two leverage the robot's structural adaptability and exceptional extension‐to‐contraction ratio (7:1, about 3.5 times that of a human hand) to enable functions beyond human hand limits (bottom of Figure 1C).

Owing to this functional biomimetic design, the BioflexBot achieves an exceptional range of graspable objects spanning over 2300 times its minimum scale, outperforming most existing dexterous hands and grippers with comparable actuation inputs by more than 12.9‐fold, as illustrated in Figure 1D. In this study, BioflexBot demonstrates its versatility across various automated grasping scenarios. Furthermore, it transcends the physiological limitations of the human hand, with promising applications such as endoscopic inspection of aeroengine blades. Importantly, the system offers a lightweight, cost‐effective, and multifunctional alternative to bulky and expensive humanoid robotic hands, enabling simpler integration, broader adoption, and lower computational burden for control. This work establishes a new paradigm for robotic end‐effectors characterized by four “S” principles: Simple in Actuation, Smart in Embodied Function, Sensible in cost, and Supreme in performance. Its versatile design unlocks applications in autonomous laboratories, endoscopic inspection, and agile service robotics, while advancing scalable and robust robotic manipulation across diverse industries.

## 2 Results

### 2.1 Functional Biomimetic Design Methodology

To construct the BioflexBot's structure, we first analyzed and classified the mechanical principles underlying four common human hand functions [13, 14, 15, 16] and identified four additional capabilities beyond what a single human hand can achieve. Building on this, we designed the overall structural configuration of the BioflexBot and investigated its deformation behaviors under various actuation modes to meet these functional requirements.

#### 2.1.1 Decomposition of Human Hand Functions and Mechanical Principles

Following a functional bionics design methodology, we systematically analyze and classify human hand grasping functions and establish functional‐to‐mechanical relationships. Since the human hand comprises fingers and a palm, its dexterity primarily arises from the interaction between fingers or the coordination between fingers and the palm [17, 18]. Therefore, we categorize hand functions into the following four types:

1. *Finger Pinching (F‐pinch)*: Objects are pinched via compressive forces generated by the interaction between 2 to 3 fingers, typically involving small and discrete contact areas [19, 20].
2. *Rotating*: Achieved through a couple moment generated through 2 or 3 fingers applying friction forces, enabling the manipulation of objects like screwdrivers, bottle caps, etc.
3. *Hooking*: It usually involves bending 4 fingers together to hook an object, with the weight of the held item distributed across every finger.
4. *Finger‐Palm Grasping (FP‐grasp)*: Objects are grasped through compressive forces resulting from the coordination of all fingers, the palm, and the object, allowing for firm grasping over larger surfaces, such as an apple or bottle.

#### 2.1.2 Defining Capabilities Beyond the Human Hand

- *Large‐Scale Grasping*: The limited extension‐to‐contraction ratio of the human hand restricts its ability to perform large‐scale grasping with a single hand [45, 46].
- *Long‐Distance Operation*: Tasks involving long‐distance manipulation, such as pushing or pulling drawers, depend heavily on coordinated movement of the entire arm [47, 48].
- *Confined‐Space Delivery*: Human hands face significant challenges when operating in confined spaces, restricted by the intricate structure of the musculoskeletal system, such as retrieving target objects through narrow gaps.
- *Multi‐Object Sequential Transportation*: Multi‐object manipulation is challenging, and transporting items sequentially is even more demanding [49, 50]. Humans typically accomplish this through coordinated bimanual and arm movements, grasping several items and placing them one by one in different locations.

#### 2.1.3 Structure Design of BioflexBot

Guided by the outlined functional requirements, the BioflexBot employs pneumatic flexible fabric air pouches as its primary mechanism. Pneumatic systems offer multiple advantages over other actuation methods [51], but the inherent symmetrical expansion of air pouches during inflation limits their ability to perform the asymmetric deformations required for complex manipulation. To address this, we integrate a flexible coil spring as a skeleton structure, as shown in Figure 1B. By leveraging the antagonistic interaction between the air pouch's expansion force and the restoring force of the coiled spring, we introduce asymmetrical deformation into the structure. The multi‐layer coiled spring design also enables a large extension‐to‐contraction ratio, approximately 7:1, and its elongation is accompanied by rotational movement at the robot's distal end.

The fabrication process begins by pre‐stretching the coil spring to flatten it, followed by bonding the spring and the TPU fabric air pouch using a heating press machine. After cutting the spring to release the stretch force, the BioflexBot is obtained efficiently. We further investigated the effect of the relative positioning of the coiled spring within the air pouch on deformation behavior under pneumatic actuation.

We identified multiple structural configurations to enable distinct deformation modes. When the flexible skeleton is aligned along the upper surface of the air pouch (Text S1), inflation begins at the air inlet and progresses distally, causing sequential flattening, longitudinal extension, rotation, and bending at the distal end during inflation. To maintain symmetry along the width and avoid deformation, the flexible skeleton of the robot is uniformly distributed across the upper surface. We defined this deformation mode as Mode 1 (Extension Mode), and the air pouch leading to upper skeleton placement in this configuration referred to as APU. In contrast, placing flexible skeletons bilaterally on both sides of the air pouch results in symmetrical, outward radial expansion upon inflation (Figure 1B bottom). This is Mode 2 (Expansion Mode), and the air pouch with lateral skeleton placement is called APL.

The two modes demonstrate versatile deformation capabilities, including elongation, rotation, and expansion, supporting core hand functions and some beyond human capabilities. However, these modes still face limitations in the precision pinching of fine objects. Experimental observations (Figure S2A) reveal that during initial inflation in expansion mode, the BioflexBot simultaneously exhibits inward contraction and outward expansion, compressing the internal cavity as the external radius increases. Upon reaching a critical compression point, further inflation intensifies external expansion while the internal cavity reopens, preventing full closure and limiting pinching force (grey curve, Figure S2C). To address this, we introduced a constraining outer shell that suppresses radial expansion and enables internal space closure (Figure S2B), thereby allowing effective gripping of microscale objects (red curve, Figure S2C). We define this as Mode 3 (Contraction Mode), capable of replicating the F‐pinch function.

In summary, the BioflexBot architecture integrates two flexible skeletons, a dual‐chamber system (APU and APL) together with a constraining outer shell. APL features symmetrically distributed skeletons on both sides, while APU positions the skeleton on the upper surface for extension mode. This design requires only two pneumatic inputs to achieve multiple deformation modes and a range of manipulations, from replicating to surpassing human hand functions. Specifically, inflating the APU triggers extension mode, while inflating APL with or without the constraining shell enables contraction mode or expansion mode, respectively. In sum, these modes can produce diverse dexterous deformations, including elongation, rotation, bending, expansion, and contraction, offering a wide spectrum of functional capabilities.

### 2.2 Structural Parameters Optimization and Deformation Characterization

Building on the previous analysis of how the coiled spring's position affects deformation, we found that the air pouch's dimensional parameters critically influence each mode's deformation behavior. This section focuses on optimizing these parameters to improve the BioflexBot's graspable size range and control precision. To determine suitable air pouch dimensions, we first selected a coil spring with a thickness of 0.2 mm as the baseline choice. Thicker springs tend to undergo greater plastic deformation under the same curvature change, which can reduce fatigue life, whereas thinner springs provide insufficient restoring force, thereby limiting the maximum load that can be grasped. Based on this preliminary selection, we further conducted expansion tests to evaluate the performance of the air pouch.

**FIGURE 2:**
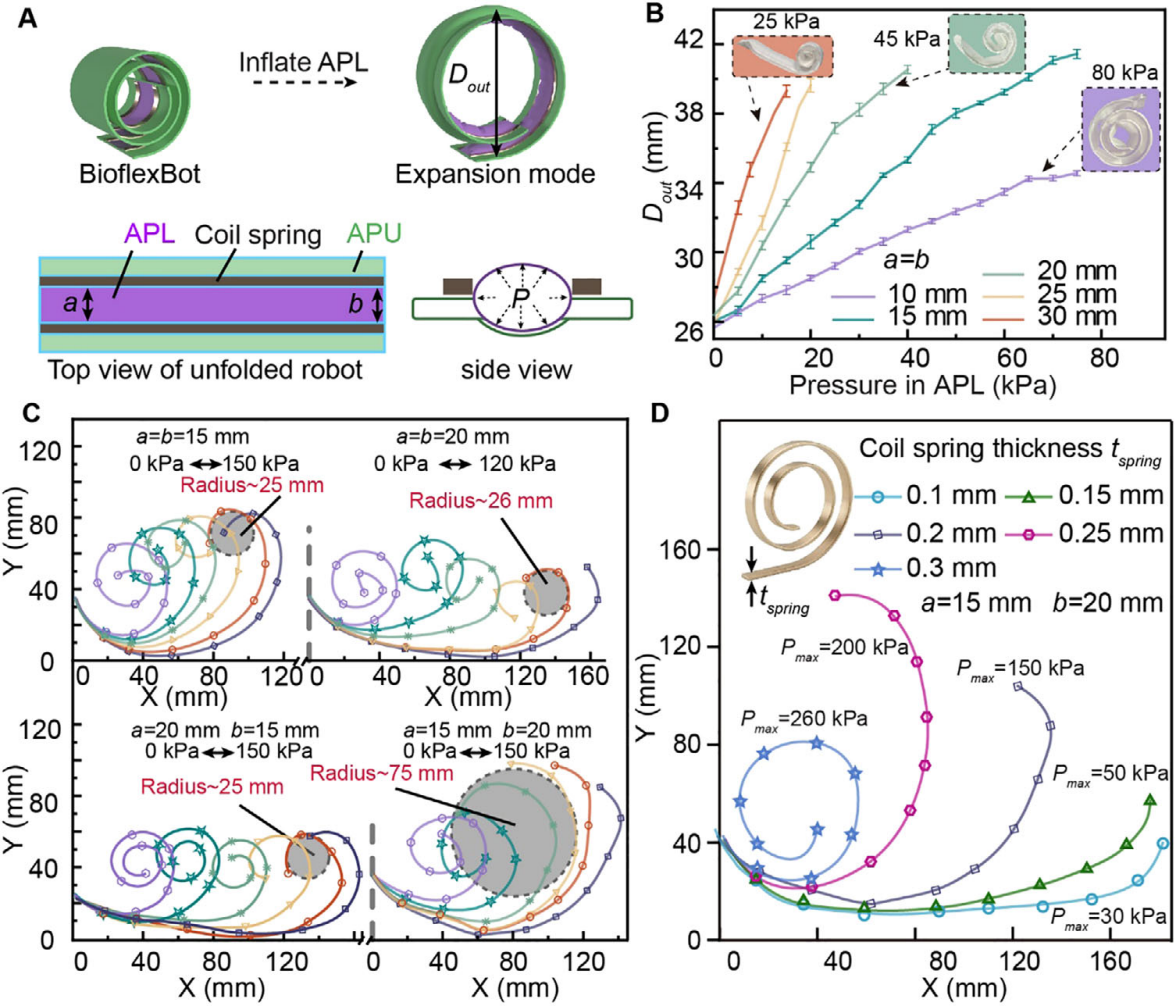
Actuation performance characterization and structural parameter optimization of the BioflexBot in expansion mode. (A) Parametric representation of the BioflexBot during expansion mode by inflating the APU. (B) Expanded diameter as a function of air pressure for different APU chamber widths. (C) Morphological changes of the BioflexBot with increasing pressure under varying APL chamber widths. (D) Maximum expansion configurations and the corresponding peak pressures required to achieve them for different coil spring thicknesses, with geometric parameters a and b set to 15 and 20 mm, respectively.

As shown in Figure 2A, the lengths of both APU and APL match the original length of the coiled spring, modeled as an Archimedean spiral defined by inner diameter, outer diameter, and coil tightness. For effective pinching combined with sufficient extension, we selected an inner diameter of 15 mm and an outer diameter of 30 mm, resulting in a spiral length of 210 mm, seven times the contracted diameter. Analysis reveals that APU's width (blue in Figure 2A) and APL's width (purple) are interdependent; however, variations in APU width have minor effects on extension mode. In contrast, expansion and contraction modes strongly depend on APL width, directing our optimization efforts there.

We tested APL widths from 10 to 30 mm in 5 mm increments under varying pneumatic pressures (Figure 2B). Narrow widths (10 mm) show steady radial expansion (expansion rate δ*D*/δ*P* ≈ 0.12 mm/kPa) up to 65 kPa, after which the expansion rate drops sharply. Wider chambers (30 mm) transition from radial expansion to tangential elongation at low pressure (∼15 kPa), due to stronger extension forces overcoming spring restoration, inducing a premature mode switch (Figure S3 and Text S2). Narrow chambers lack sufficient extension force, limited instead by interlayer compression of the air pouch. To avoid insufficient expansion or early transition, we designed APL with a trapezoidal cross‐section (bases 15 and 20 mm), balancing uniform expansion and efficient deployment. Vision‐based motion capture experiments (Figure 2C) were conducted to compare trapezoidal and rectangular chamber designs. The results show that the APL configuration, with parameter a = 15 mm and b = 20 mm, exhibits a wider C‐shaped deformation, enabling the robot to grasp objects up to approximately 75 mm in diameter, which is three times larger than those achievable with the other three designs, as illustrated in the lower right corner of Figure 2C.

With the air pouch dimensions fixed, we further investigated the effect of coil spring thickness on the expansion mode. The experimental results (Figure 2D) show that when the coil spring is too thin (0.1 or 0.15 mm), the BioflexBot fully expands under low pressure, thereby limiting the maximum weight that can be grasped. In contrast, when the spring thickness is 0.3 mm, the robot cannot fully expand even at pressures up to 260 kPa. As for the spring thickness of 0.25 mm, although it achieves a comparable expansion performance to that of 0.2 mm, it requires a substantially higher pressure of up to 200 kPa. Such elevated pressure levels can accelerate the onset of air pouch rupture. Therefore, for the selected air pouch dimensions, a coil spring thickness of 0.2 mm represents the optimal choice.

For the APL, we also theoretically modeled the maximum graspable sizes corresponding to different deformations induced by varying pressures. First, we assume that the inflated BioflexBot forms an arc segment with radius *R*, corresponding to a central angle α. We further assume that any grasped object can be approximated by its circumscribed rectangle. Finally, we assume an object is considered graspable if its circumscribed rectangle lies entirely within the enveloping surface formed by this arc.

When γ < π, for any rectangle length satisfying *l*  =  2*R*sinβ, where β∈(0,γ/2), the geometric relationship (as shown in Figure S4A) indicates that only two points lie on the curve, yielding its corresponding maximum width *w* as:

(1) $$w_{max}=R\cos\beta -R\cos\left(\frac{\gamma }{2}\right)$$

When γ > π, the grasped rectangle may have two or four corner points in contact with the arc. For the two‐point contact scenario (Figure S4B), its length *l* satisfies the relationship *l*  =  2*R*sinβ, where β∈(0,π−γ/2). In this case, the corresponding maximum width *w* can be expressed as:

(2) $$w_{max}=Rcos\left(\pi -\frac{\gamma }{2}\right)+Rcos\beta =Rcos\beta -Rcos\left(\frac{\gamma }{2}\right)$$

For the four‐point contact scenario (Figure S4C), its length *l* satisfies the relationship $l=2R\sin\beta ,\beta \in (\pi -\frac{\gamma }{2},\frac{\gamma }{2})$. In this case, the corresponding maximum width *w* can be expressed as:

(3) $$l^{2}+w^{2}=R^{2}$$

Directed by this model, we further experimentally verified the maximum graspable size of objects using the BioflexBot, which will be introduced in detail in next section.

**FIGURE 3:**
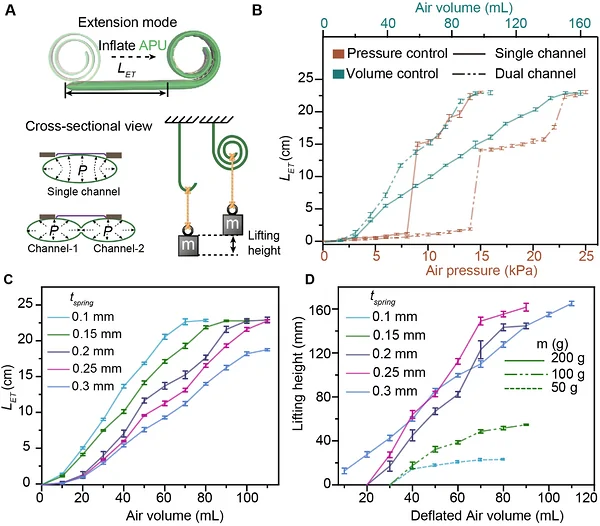
Channel structure optimization for extension mode and characterization of actuation performance. (A) Schematic illustration of the robot operating in extension mode, highlighting its key geometric parameters. (B) Effects of single chamber versus dual chamber configurations and pressure control versus volume control actuation on the robot's extension length. (C) Relationship between inflation volume of the APU and the resulting extension length for robots with different coil spring thicknesses. (D) Relationship between the released air volume from the APU and the achievable lifting height for robots with different coil spring thicknesses. Robots with 0.1 and 0.15 mm thick coil springs, whose stiffnesses are low, exhibit limited load capacities, with the maximum lifting masses of 50 and 100 g, respectively.

For APU, excessive vertical expansion during extension mode led us to split it into two interconnected channels (Figure 3A) to constrain the expansion height. Comparative tests (Figure 3B) between single‐ and dual‐channel APU under pneumatic pressure showed abrupt elongation transitions in both, which lowered control precision due to fluid viscosity‐chamber interaction. By switching to volumetric (air volume) control, extension became more uniform, and the dual‐channel APU required significantly less air. Based on these results, the final BioflexBot structure features an APL with a trapezoidal channel and symmetrically distributed flexible skeletons along its sides, alongside a dual‐channel APU with uniform upper surface skeleton placement. Volume‐based control is employed for the APU to enhance precision and efficiency.

Finally, to verify that the selected coil spring thickness is also suitable for the extension mode, we experimentally evaluated the effects of different spring thicknesses on the inflation–extension relationship (Figure 3C) and the lifting height as a function of deflated air volume (Figure 3D). The results indicate that thinner springs require less injected air volume to achieve the same extension length. For a spring thickness of 0.3 mm, the force generated by the air pouch is insufficient to fully straighten the spring, resulting in a slightly curved configuration; consequently, its measured maximum extension length is smaller than that of the other groups. In addition, for a given object, the achievable lifting height increases with spring thickness. Consistently, the 0.3 mm spring exhibits a relatively limited lifting height due to its residual curvature. For the 0.1 and 0.15 mm springs, the maximum liftable masses are 50 and 100 g, respectively. Based on these results, 0.2 mm can be identified as the optimal coil spring thickness for this study.

### 2.3 Bio‐Functional Mimicry Characterization and the Reliability Tests of BioflexBot

Based on the optimized design, we fabricated a prototype and conducted systematic experiments to characterize and validate the functions. Tests were organized according to the complexity of finger and palm cooperation: starting with finger pinching (F‐pinch) and rotation involving 2–3 fingers, progressing to hooking involving 4 fingers, and finally finger‐palm grasping (FP‐grasp) involving all five fingers and the palm.

#### 2.3.1 F‐Pinching

**FIGURE 4:**
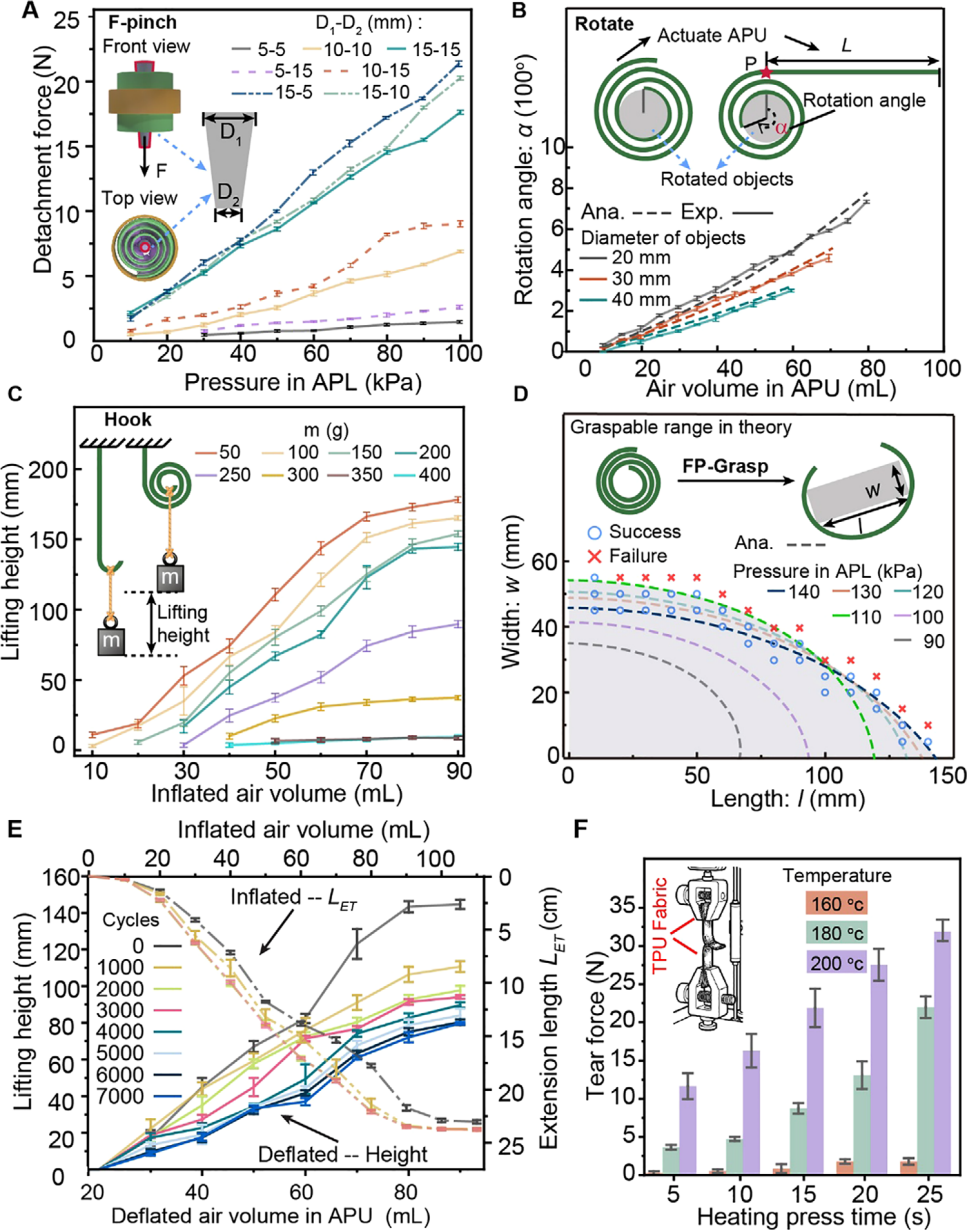
Experimental characterization of BioflexBot's bio‐functional mimicry and its reliability tests. (A) Detachment force versus air pressure during F‐pinch for objects of different sizes. (B) Relationship between rotational angle and object diameter under varying input air volumes, which is validated experimentally. (C) Lifting height as a function of input air volume for objects with different masses. (D) Comparison between theoretical predictions and experimental results for the maximum graspable object size across varying pressures in the FP‐grasp mode. (E) The robot's extension length under inflated air volume and the achievable lifting height of objects during deflated air volume during the repeated inflation–deflation cycles (up to 7000 cycles). (F) Effects of heat‐pressing temperature and time on the maximum tearing force sustained by the heat‐pressed seams during the robot's thermal pressing process.

**FIGURE 5:**
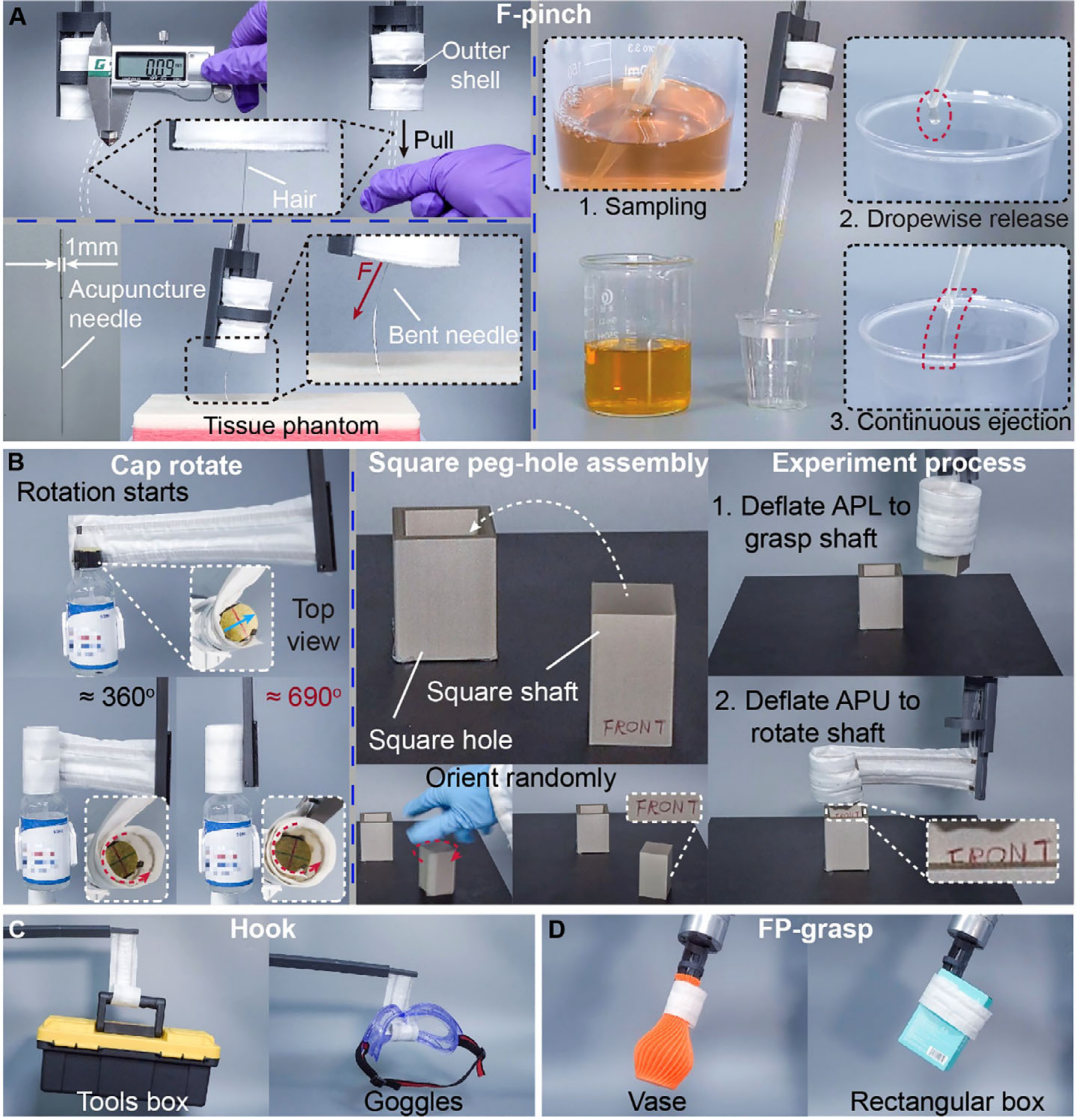
Demonstration of BioflexBot's functions via bio‐functional mimicry in human‐like grasping. (A) Precision manipulations enabled by the F‐pinch mode: stable handling of a human hair, insertion and removal of an acupuncture needle in a tissue phantom, and precise liquid transfer using a pipette. (B) Rotation function demonstrated by unscrewing a bottle cap and performing orientation‐based assembly of a square peg into a hole. (C) Successful hooking of objects with different shapes and sizes, showcasing configurational adaptability. (D) Secure FP‐grasping of various objects with different geometries.

Prior characterization showed that the addition of a constraining shell around the APU significantly enhances F‐pinch capability. We verified this through pressure‐loading and geometry tests on 3D‐printed cylinders and conical frusta whose diameters vary from 5 to 15 mm (Figure 4A). Experimental results for grasping objects of the same shape indicate that the maximum pull‐off force increases with the object's diameter. For conical frustum objects, the maximum pull‐off force for an inverted frustum (D_1_ > D_2_) is significantly higher than that for an upright frustum (D_1_ < D_2_). This difference arises from the self‐adaptive nature of the gripper. Its grasping force is always applied perpendicularly to the object surface, thereby producing an effective component along the pull‐off direction (Figure S5). As a result, the inverted frustum can withstand a larger pull‐off force. It is also noted that, for the upright frustum (10 mm–15 mm), the effective radial dimension at the contact region exceeds 10 mm; therefore, its maximum pull‐off force remains higher than that of a cylindrical object with dimensions of 10 mm × 10 mm. We then evaluated cross‐scale adaptability and force controllability of F‐pinch across scales from standard objects (pens, straws), as shown in Figure S6A and B, to microscale targets such as a 90 µm human hair (Figure 5A and Movie S1). The BioflexBot reliably pinched objects up to its 15 mm internal diameter. To test force control precision, two application‐driven experiments were conducted: Medical procedure simulation: Acupuncture needle insertion into a tissue phantom was performed by pinching a 1.1 mm needle tip, gradually increasing pinch force to prevent slippage during insertion and extraction (the left panel of Figure 5A and Movie S1 and Figure S7). Chemical liquid transfer: The BioflexBot manipulated a pipette tip to sample, lift, and dispense liquids via carefully controlled pressure ramps, demonstrating precise force modulation suitable for laboratory work (the right panel of Figure 5A and Movie S1 and Figure S8). These results validate BioflexBot's capability for fine force control, which is essential in delicate tasks.

#### 2.3.2 Rotating

The human hand rotates objects by applying a couple moment with fingers generating frictional torque, such as when turning a cap or screwdriver. Similarly, the BioflexBot produces self‐rotation around its center during extension mode by actively inflating the APU, enabling it to exert rotational moments on objects. To investigate this relationship, we analyzed the correlation between APU inflation volume, rotation angle, and object diameter. First, based on the geometric analysis of the BioflexBot, we established a kinematic model, as shown in Figure S9, with the corresponding Equations (S1–S3) provided in Text S4. Next, a polynomial fitting method was adopted to describe the relationship between the APU inflation volume and the elongation length (Equation S4). By combining these Equations, the model can theoretically predict the rotation angle for objects of different diameters under varying inflation volumes. It should be noted that, because air is compressible, precisely controlling the inflation volume is challenging. To improve accuracy and repeatability, we developed a lead‐screw‐driven syringe platform. In each experiment, the initial air volume in the cylinder at atmospheric pressure was set equal to the target inflation volume of the APU. During inflation, this entire volume was fully delivered into the APU. Therefore, in this study, the controlled input variable is defined as the volume of air at atmospheric pressure delivered into the chamber, rather than the final air volume inside the chamber after inflation. Based on this definition and theoretical analysis, we conducted validation experiments. As shown in Figure 4B, the experimental results for objects of various diameters were consistent with the model predictions, confirming the model's effectiveness in enabling controllable rotation.

Building on this, we demonstrated practical rotation tasks such as bottle cap manipulation (the left panel of Figure 5B and Movie S2) and shaft‐hole assembly (the right panel of Figure 5B and Movie S2). The cap unscrewing procedure involved fully extending the BioflexBot beside the cap via APU inflation, then controlled deflation to envelop and rotate the cap counterclockwise through axial contraction. The BioflexBot achieved up to 690° rotation in one continuous motion, which is nearly four times the typical human hand's single‐turn angle (180°). Re‐inflation reversed rotation to tighten the cap reliably. In the square peg–hole assembly, the hole was randomized manually, the peg grasped by controlling APL inflation, and the peg rotated by inflating APU until correctly aligned. Coordinated with a robotic arm, this demonstrated precise directional rotation for polygonal objects, highlighting BioflexBot's versatility in rotating both circular and angular items through controlled extension.

#### 2.3.3 Hooking

The BioflexBot utilizes its extension mode to perform hooking. Building on prior findings that correlated unloaded extension length with APU inflation volume, we experimentally examined the relationships among extension length, lifted weight, and inflation volume. As shown in Figure 4C, for a fixed inflation volume, lighter objects achieve greater lifting heights and maximum elevation. For a given object mass, increasing deflation volume further raises the lifting height, though with diminishing returns due to the coiled spring's restoring force decreasing as its curvature reduces during lifting.

We then tested the hooking performance (Figure 5C and Figure S10 and Movie S3), revealing the operational sequence: Inflate APU to extend the BioflexBot with a bent tip; Bring the bent tip into contact with the target object; Partially deflate the APU to contract and helically wind around the object, securing a tight grip. These experiments demonstrate that the BioflexBot can hook both annular objects (e.g., toolbox handles, headphone arcs) and protruding items (e.g., goggles frames, slotted containers).

#### 2.3.4 FP‐Grasping

In everyday tasks involving large objects such as cups and apples, stable grasping typically requires coordinated force from both the fingers and palm. Leveraging prior optimization, the BioflexBot exploits its expansion mode to achieve a wide unfolding span, effectively mimicking the human hand's grasping mechanism. This enables secure manipulation of large objects. To evaluate the BioflexBot's grasping capacity, we developed a theoretical model to predict graspable object dimensions. Figure 4D compares experimental data with model predictions, showing strong agreement with the predicted maximum graspable size. The robot's dynamic gripping forces were tested and recorded under varied inflation and deflation pressure, as shown in Figures S11 and S12. Finally, comprehensive demonstrations of the FP‐grasp mode (Figure 5D and Figure S13 and Movie S3) confirm that the BioflexBot can securely handle various daily objects, including vases, fruits, and boxes, which highlights its practical versatility and adaptability for complex tasks.

#### 2.3.5 Reliability Test

Because air leakage and fatigue of the coil spring will significantly influence the robot's performance, a cyclic testing platform was fabricated. A photoelectric sensor and a counter were used to measure the extension length during the cyclic test. The air leakage phenomenon, the relationships between extension length and inflated air volume, and the relationship between achievable lifting height (a 200 g load) and the deflated air volume were tested and recorded every 1000 complete cycles of inflation and deflation, with results shown in Figure 4E. For the issue of the coil spring's fatigue, the results indicate that fatigue significantly affects the extension behavior during the first 2000 cycles. For a given inflation volume, the extension length increases noticeably during the first 2000 cycles, compared with that of the robot in its initial state. From 3000 cycles on, the extension length exhibits approximately the same trend and values, suggesting that fatigue effects stabilize after approximately 2000 cycles. In terms of achievable lifting performance, the achievable lifting height gradually decreases with increasing cycle number and levels off after around 5000 cycles. After testing for more than 7000 cycles, the coil spring fractured, for which reason the extension length and lifting height test was terminated at this number of cycles.

However, the air leakage test was extended beyond 7000 cycles to 10000 cycles, during which no detectable air leakage was observed, demonstrating the robot's durability under practical operating conditions. We further evaluated its robustness by intentionally inducing damage, including tearing the heat‐pressed seams and puncturing the membrane. These leakage issues can be rapidly repaired by reapplying heat pressing to the seams or attaching a patch to the damaged membrane. After repair, the robot was tested again and was able to sustain deformation over several hundred additional cycles, indicating good recoverability.

In addition to air leakage and fatigue under cyclic inflation and deflation, the tear resistance of the heat‐pressed seams is another critical factor affecting the reliability of the air pouch. Tearing tests (Figure 4F) indicate that seam strength is positively correlated with both heat‐pressing temperature and duration. Specifically, heat pressing at 200°C for 25 s produces a maximum tearing force of approximately 31 N. By adjusting the fabrication parameters, a wide range of bonding performance can be achieved, with the minimum tearing force as low as 0.27 N at 160°C for 5 s.

### 2.4 Functional Demonstration of an Upper Limb With Adaptive Growing Ability

Prior experiments have validated the effectiveness of the BioflexBot in replicating common human grasping functions. Moving beyond simple mimicry, the BioflexBot leverages coordinated extension and contraction modes to function as an upper limb with adaptive growing ability. This enables manipulations that are challenging or even impossible for a single human hand. This section highlights several such capabilities that exceed human hand performance.

#### 2.4.1 Large‐Scale Grasping

**FIGURE 6:**
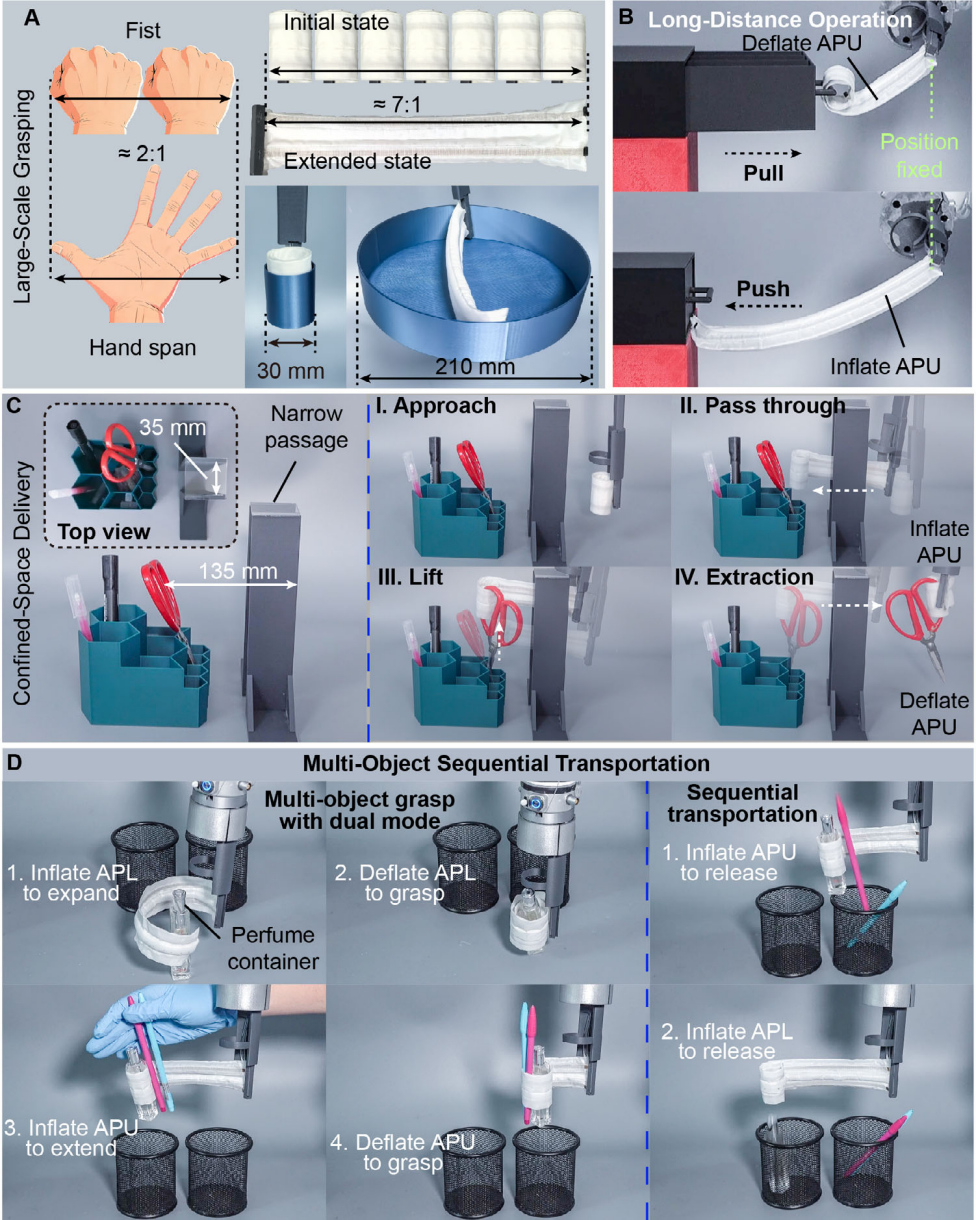
Demonstration of BioflexBot functioning as an upper limb with adaptive growing capability. (A) Large‐scale grasping: The robot achieves a graspable range approximately seven times its initial size. (B) Remote operation: Leveraging its adaptive growing ability, the robot performs long‐distance operations, such as pushing and opening a drawer without repositioning its base. (C) Confined spaces delivery: Enabled by its over 7:1 extension‐to‐contraction ratio, the robot reaches and grasps objects within narrow channels. (D) Multi‐object sequential transportation: Coordinated extension and expansion modes allow the robot to sequentially grasp and sort objects of varying sizes.

Human hands are typically limited to motions toward the palm, confining their effective workspace to roughly twice the size of a fist span [52, 53]. The BioflexBot uniquely achieves large‐scale supportive grasping through its expansion mode. Thanks to its high extension‐to‐contraction ratio of 7:1, the BioflexBot can grasp objects with internal cavities ranging from 30 to 210 mm in diameter (Figure 6A). To further evaluate adaptability, we tested grasping of objects with variously shaped inner surfaces. Experimental results (Figure S14 and Movie S4) show that even with complex cavity geometries, the BioflexBot maintains excellent conformity to shapes, underscoring its versatility in handling a wide variety of internal structures and sizes.

#### 2.4.2 Long‐Distance Operation

Due to anatomical constraints, the human hand relies heavily on arm coordination for reaching distant objects. The BioflexBot surpasses this limitation with its remarkable deformation capability and extension‐to‐contraction ratio of 7:1, exceeding the typical arm length to hand‐palm size ratio of about 3:1 [54]. Capitalizing on this, the BioflexBot operates as a remote manipulation system integrated with a robotic arm, demonstrated by precision drawer opening and closing (Figure 6B and Movie S4). The process involves: fully inflating the APU to extend the BioflexBot and guiding its tip to the drawer handle via a robotic arm; fixing the arm's pose; controlled deflation of the APU to contract the BioflexBot and pull the drawer open; then re‐inflating the APU to extend the BioflexBot and push the drawer closed, all without changing the robotic arm's base position. This experiment highlights the BioflexBot's capacity for remote grasping and manipulation, enabling extended reach and dexterity beyond the limits of a single hand.

#### 2.4.3 Confined Space Delivery

Benefiting from its compact contracted diameter of 30 mm, the BioflexBot can pass through narrow openings and perform grasp‐and‐retrieve operations via its extension mode. To validate this, we designed a slit traversal and scissor grasping experiment (Figure 6C and Movie S5). The procedure is as follows: 1) Position the contracted BioflexBot at the mid‐point of a 35 mm‐wide slit (just above its outer diameter), then inflate to extend through the gap and approach scissors located 135 mm away; 2) Deflate the APU to grasp the scissors securely, then lift to extract them from the container; 3) Continue controlled deflation to bring the scissors safely back through the slit. Beyond simple linear passages, the BioflexBot also demonstrates passive adaptability within complex cavities. For example, when encountering obstacles from cavity walls during elongation, it passively adjusts its path to navigate curved passages successfully. This adaptive traversal and grasping were demonstrated in a simulated complex piping environment (Figure S15 and Movie S5), confirming the BioflexBot's capability to maneuver in unstructured spaces.

#### 2.4.4 Multi‐Object Sequential Transportation

Tasks involving multi‐object sequential transportation typically require the coordination of both hands and the arm. The BioflexBot leverages its planar spiral structure to grasp multiple objects simultaneously through interlayer compression during contraction, allowing controlled, sequential release through gradual extension to perform multi‐object transportation. To validate this, we designed an experiment (Figure 6D and Movie S6) where the BioflexBot simultaneously grasps a large‐diameter glass container and a small pen, then deposits them into two separate containers in sequence. The procedure includes: 1) Inflating the APL channel to deform the BioflexBot, then deflating to envelop the glass container using FP‐grasping; 2) Inflating the APU channel to initiate extension mode but stopping before full extension to hold the glass; 3) Deflating the APU to contract and grasp the pen; 4) Inflation of the APU (to sequentially release the pen) followed by inflation of the APL to release the glass container, completing the transportation sequence. To further test sequential manipulation, we grasped five pairs of chopsticks arranged left to right, selectively placing required numbers into different containers (Figure S16 and Movie S6). These experiments confirm BioflexBot's effectiveness for sequential multi‐object handling and demonstrate the compatibility of its dual‐channel control scheme, significantly expanding its functional range.

### 2.5 Functional Demonstration of Tool‐Hand Integration

The proposed BioflexBot's lightweight architecture and simple configuration make it highly adaptable as an independent module, suitable for integration into various systems. This section experimentally explores two integration approaches and their applications: 1) self‐integration of multiple BioflexBots as an aeroengine blade array inspection tool, and 2) integration with a humanoid robot as a lightweight, multifunctional hand.

**FIGURE 7:**
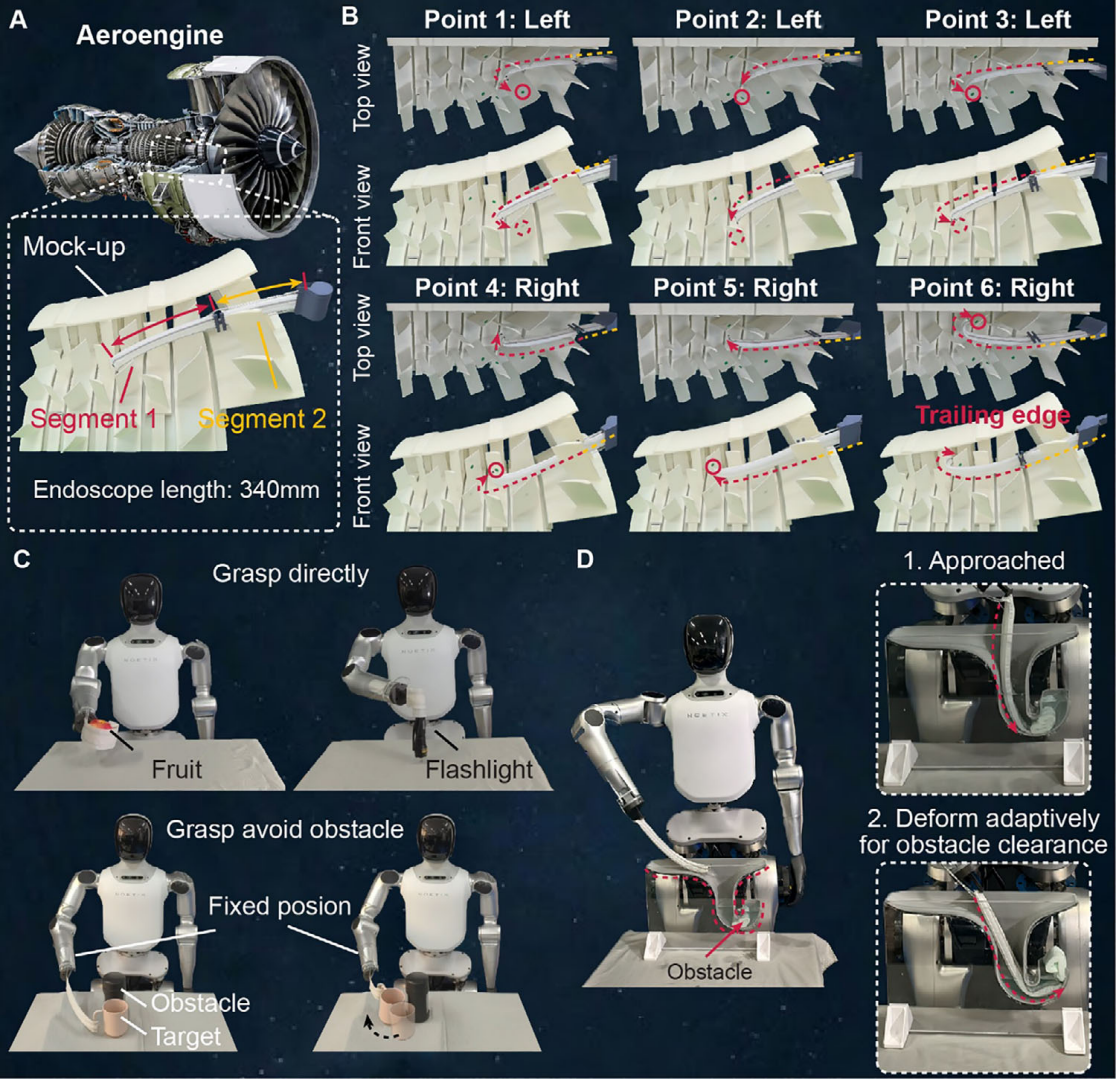
Demonstration of BioflexBot leveraging its tool‐hand integration capability. (A) Inspection of an aeroengine blade array performed by the BioflexBot. The 3D‐printed mock‐up of the engine and the experimental setup. (B) Inspection process of the blade arrays. Notably, the robot's large‐curvature distal bending enables successful inspection of the blade's trailing edge (Point 6: right). (C) Experimental demonstration of BioflexBot serving as a humanoid robot's hand for direct grasping and obstacle‐avoiding grasping. (D) Clearing obstructions within a narrow channel utilizing the BioflexBot.

As illustrated in Figure 7A, this study conducted inspection experiments using a 3D‐printed, 1:1 scale mock‐up of aeroengine blade arrays. In this study, two BioflexBot modules were connected in series, forming a self‐growing endoscopic robot. The proximal module uses elongation mode to inspect nearby regions, while the distal module elongates to self‐propel the proximal module deeper into the engine. The inspection outcomes (Figure 7B and Movie S7) cover six points on both sides of the engine blade channel. Notably, the system's large‐curvature distal bending enables successful inspection of the trailing edge of a blade (Point 6: right), a challenging task for traditional methods [55, 56, 57].

In another application, the BioflexBot serves as a lightweight, cost‐effective, and multifunctional alternative to dexterous hands for humanoid robots. As shown in Figure 7C and Movie S8, a humanoid robot successfully completed various daily grasping tasks using the proposed BioflexBot, including direct grasping of fruits and tools, and obstacle‐avoidance grasping tasks. Notably, in the task of grasping a cup bypassing an obstacle without changing the robotic arm's pose, enabled by the BioflexBot's high extension‐to‐contraction ratio. This capability effectively reduces the control complexity and power consumption of the robotic system. Additionally, leveraging this high extension‐to‐contraction ratio, the humanoid robot equipped with the BioflexBot can reach into confined pipelines to clear blockages (Figure 7D), a task that cannot be performed by conventional humanoid hands due to workspace limitations. These experiments demonstrate BioflexBot's feasibility in the function of too‐hand integration. Its lightweight and elegant design, ease of control, and multifunctionality provide excellent potential for integration with various robotic platforms, laying a foundation for expanding its range of applications in the future.

### 2.6 Demonstration of BioflexBot's Integrated Multifunctionality

**FIGURE 8:**
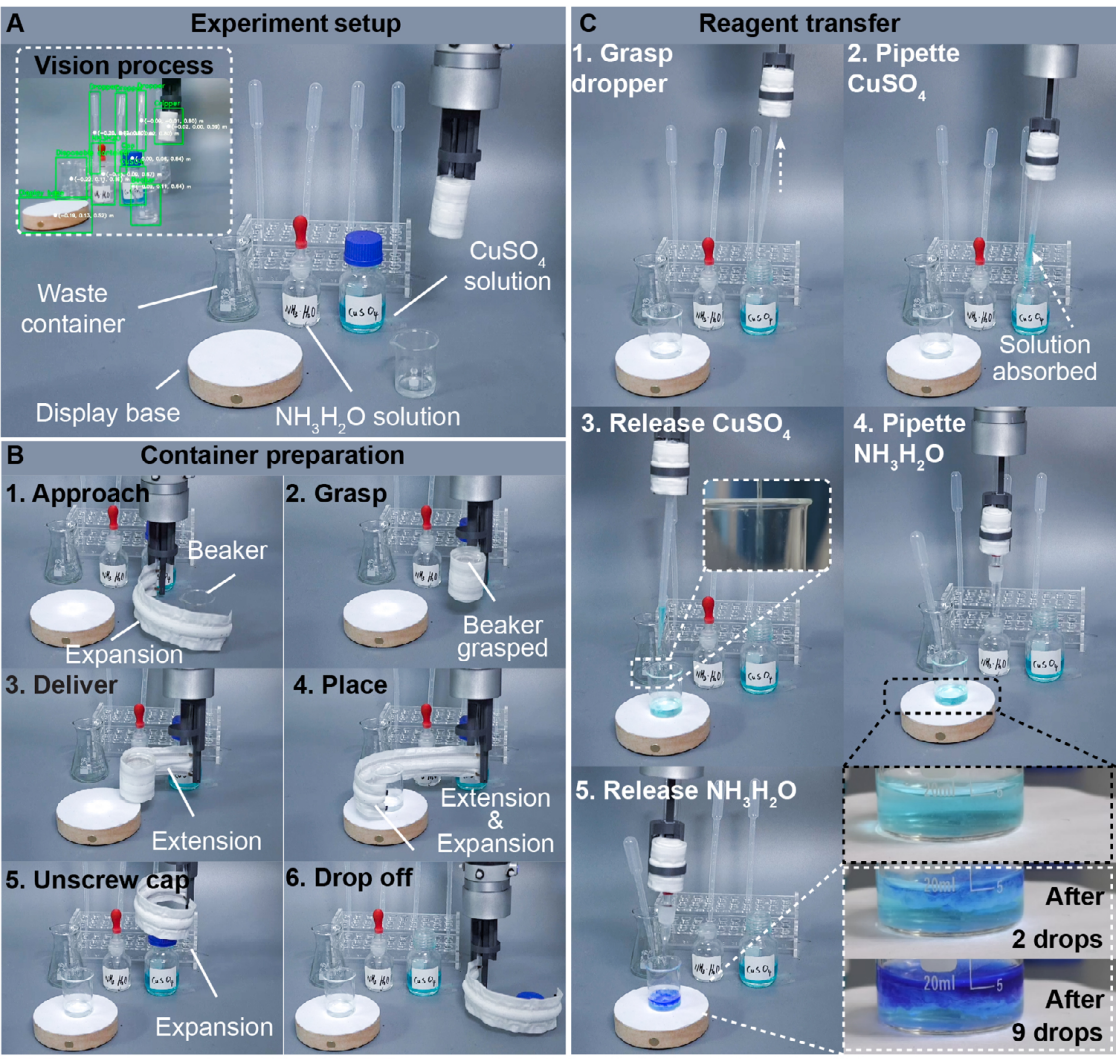
Demonstration of an automated chemistry experiment utilizing the coordinated multifunctionality of BioflexBot. (A) Setup of the automated chemistry platform, featuring vision‐based target localization and status monitoring. (B) Grasping and precise placement of the container for subsequent chemical processing. (C) Controllable transfer of reagents and visualization of the resulting chemical reactions.

Building on the thorough investigation of individual functions, the diverse capabilities of the BioflexBot have been validated. To further examine the compatibility and effectiveness of multifunctional coordination in realistic, complex scenarios, we designed an integrated chemistry experiment, as illustrated in Figure 8. This experiment leverages multiple functional modes of the BioflexBot.

To emphasize the potential for automated control, a binocular vision system paired with a YOLOv8‐based deep learning algorithm (Top left of Figure 8A) was integrated. This setup enables real‐time recognition and localization of laboratory apparatus. Coupled with a pre‐trained optimal robotic arm motion trajectory, it supports intelligent perception and decision‐making, allowing the BioflexBot to execute laboratory tasks in an autonomous and precise manner. The full operation sequence and outcomes are detailed in Figures 8B,C, and Movie S9. The procedures of the experiment are divided into two main phases: beaker placement and solution sampling.

Beaker Placement and Cap Unscrewing (Figure 8B): 1) The BioflexBot approaches the beaker containing water for solution dilution. 2) Using FP‐grasping, the BioflexBot securely grips the beaker. 3) The extension mode is activated by inflating the APU chamber, delivering the beaker to the presentation platform. 4) The APL channel is activated to engage the expansion mode, deploying into a stable C‐shaped configuration to stably position the beaker. 5) The rotation function is used to unscrew the cap of the water container. 6) The cap is then released by activating the expansion mode to drop it off.

Solution Sampling (Figure 8C): 1) Switching to the F‐pinching mode with the confined shell, the BioflexBot guides its tip to the rubber‐tipped dropper inside the CuSO_4_ solution bottle. 2) Inflating the APL compresses the dropper to expel internal air; subsequent deflation creates suction to draw CuSO_4_ solution inside; 3) Re‐inflating applies a moderate pinching force to securely lift and transfer the dropper while preventing solution extrusion. 4) The dropper is positioned above the beaker, and further inflation dispenses the solution precisely. 5) The dropper is replaced in its original bottle. 6) This sequence is repeated for sampling the ammonia solution.

Through these operations, the characteristic reaction between copper sulfate and ammonia was successfully observed (right corner of Figure 8C): initial formation of a light blue precipitate, followed by its dissolution and transformation into a deep blue solution as more ammonia was added. The successful completion of this chemical experiment validates the effective coordination and adaptability of BioflexBot's various functional modes in complex tasks.

## 3 Discussion

Dexterous manipulation is essential for robots to perform complex tasks across industrial, medical, and service applications. Conventional approaches pursue this capability through structural biomimicry, inevitably leading to increased mechanical complexity and control challenges. In contrast, we propose BioflexBot to challenge this by shifting design methodology from precise morphological replication to bio‐functional abstraction and implementation, demonstrating that high‐level dexterity can emerge from the design of simple mechanical elements. This approach is embodied in the Four “S” principles: Simple in Actuation, Smart in Embodied Function, Sensible in Cost, and Supreme in Performance.

Leveraging the antagonistic interaction between dual pneumatic chambers and coiled springs, BioflexBot achieves three core motions, i.e., extension, expansion, and contraction. This design strategy enables replication of multiple human hand functions with only two control channels, significantly simplifying system complexity without sacrificing capability. Beyond mimicking biological hands, BioflexBot also serves as an upper limb with adaptive growing capabilities that execute tasks inaccessible to humans, such as precise manipulation within highly confined spaces and multi‐object sequential transportation. Notably, the BioflexBot scales the graspable object size range by 12.9‐fold beyond state‐of‐the‐art systems with comparable actuation. Its inherent compliance and modular construction offer a tool‐hand integration platform for diverse, high‐impact applications. For instance, when configured as a self‐propelling endoscopic robot, it addresses the demands of navigating constrained and tortuous environments within aeroengines for blade inspection. Simultaneously, as a tool‐hand integration platform, it provides an attractive alternative end‐effector for humanoid robots, minimizing distal mass to improve dynamic stability and energy efficiency, due to its lightweight design (∼9 g). Rather than completely replacing dexterous robot hands, BioflexBot serves as a complementary, low‐cost alternative since it can access confined spaces unreachable by traditional hands and perform simple grasping tasks more efficiently to reduce system complexity and overall cost. Our robot roughly costs $5 (Table S2), while typical dexterous hands cost ranging from $500 to $50 000 (Table S3).

Despite these advances, challenges remain. Accurately predicting the complex, nonlinear deformation of the BioflexBot under pneumatic actuation requires integrating coupled fluid‐structure interaction models to establish more rigorous kinetic descriptions. Such developments are essential for enabling high‐fidelity closed‐loop control. Furthermore, transitioning from the current tethered prototype to a fully untethered, autonomous platform represents a critical next step. The integration of compact micropumps, onboard power supply, and wireless communication will render the BioflexBot a truly portable and widely deployable manipulation solution. This evolution will not only unlock new practical applications but also highlight the transformative potential of biologically inspired designs to redefine robotic manipulation, where simplicity paired with smart systems can achieve supreme functional versatility.

## 4 Materials and Methods

### 4.1 Materials and Fabrication of the BioflexBot

Materials used to fabricate the proposed robot include N70D rib weft‐knit polyester fabric with double‐sided TPU coating (0.2 mm), a coiled spring, and Ergo‐5800 adhesive. Equipment to fabricate the robot includes a heating press machine (WT‐90GS, Kajiang Co. Ltd.), a laser cutting machine (VLS 3.50; Universal Laser Systems), and a 3D printer (Ultimaker S3, Ultimaker Co. Ltd.).

### 4.2 Air Sources and Pneumatic Control System

The detailed information about hardware is as follows: an air compressor, a vacuum pump; PSOLs (Proportional Solenoid, ITV1030), and a stepper motor‐driven electric cylinder (FSK‐40J, FuYu Co. Ltd.) to regulate the pressures and air volume, respectively, which are powered by a regulated 24‐V supply (GPS305D), a microcontroller (Mega 2560, Arduino). The air pressure control also requires a digital‐to‐analog converter (DAC) module (PCF8591), reversing valves (0526T), and switching valves (0520D).

### 4.3 Equipment of Measurement and Experimental Demonstration

To collect the shape of the robot during expansion, we used the motion capture system (Mars 4H, NOKOV). This study employed a uniaxial tensile force gauge (Series, Mark‐10 Co. Ltd.) to measure the pull‐off force under grasping conditions. A robotic arm (Rm75‐6F, Realman Intelligent Technology Co., Ltd.) was employed for experimental manipulation, and a high‐resolution digital video camera (FDR‐AX700, SONY) was used to record the entire process.

## Author Contributions

X.T. and Y.T.L. conceived the concept; X.T., Y.T.L., and Y.Y. designed the methodology; X.T. and T.L.Z. conducted the device fabrication and characterization; X.T. and F.M. analyzed the model; X.T. and Q.Z. developed the code; X.T., T.L.Z, and Z.Q.S. designed and conducted robotic demonstrations; X.T. and Y.K.J. prepared resources; Y.T.L., Y.K.J., and X.T. acquired funding; X.T. and Y.T.L prepared figures and drafted the manuscript; X.T., Y.T.L., and Y.Y., revised the manuscript; Y.T.L. supervised the research.

## Funding

This study is supported by the National Natural Science Foundation of China under grant 52475038 and 62303443; and the Guangdong Basic and Applied Basic Research Foundation under grant 2026A1515012169.

## Ethics Statement

Not applicable. This study did not involve human or animal subjects.

## Consent

Not applicable. No human participants were involved.

## Conflicts of Interest

The authors declare no conflicts of interest.

## Supporting information




**Supporting File 1**: advs76527‐sup‐0001‐SuppMat.docx.

**Supporting File 2**: advs76527‐sup‐0002‐MovieS1.mp4.

**Supporting File 3**: advs76527‐sup‐0003‐MovieS2.mp4.

**Supporting File 4**: advs76527‐sup‐0004‐MovieS3.mp4.

**Supporting File 5**: advs76527‐sup‐0005‐MovieS4.mp4.

**Supporting File 6**: advs76527‐sup‐0006‐MovieS5.mp4.

**Supporting File 7**: advs76527‐sup‐0007‐MovieS6.mp4.

**Supporting File 8**: advs76527‐sup‐0008‐MovieS7.mp4.

**Supporting File 9**: advs76527‐sup‐0009‐MovieS8.mp4.

**Supporting File 10**: advs76527‐sup‐0010‐MovieS9.mp4.

## Data Availability

The data that support the findings of this study are available from the corresponding author upon reasonable request.
