# Supplementary material for: A Bio‐Functional Mimetic Robot for Versatile Tasks From Cross‐Scale Manipulation to Limb‐Tool Integration
Source: Adv Sci (Weinh). 2026 Aug 13;13(51):e76527. doi: 10.1002/advs.76527 (PMC13469783; doi:10.1002/advs.76527)
Supplement: Supplementary file 1 [file ADVS-13-e76527-s006.docx]

Supplementary Information for

A Bio-functional Mimetic Robot for Versatile Tasks from Cross-Scale Manipulation to Limb-Tool Integration

Xin Tong, Tianle Zhang, Fei Mo, Qian Zhao, Zhongqing Sun, Yongkang Jiang, Yang Yang,***** Yingtian Li*****

*Corresponding author: Yang Yang, meyang@nuist.edu.cn; Yingtian Li, yt.li@siat.ac.cn.

**This file includes:**

Supplementary Texts 1 to 5

Supplementary Figures 1 to 16

Supplementary Tables 1 to 3

Legends for Movies S1 to S9

**Other Supplementary Materials for this manuscript include the following:**

Supplementary Movies 1 to 9

Supplementary Text

1. **BioflexBot’s structure and motion modes**

The BioflexBot is composed of dual chambers and a flexible skeleton, enabling three motion modes, as illustrated in Fig. 1B. **Mode I-Extension**: Achieved by actuating the APU chambers located directly beneath the two flexible skeletons, resulting in axial elongation. **Modes II-Expansion and Modes III-Contraction:** Both are realized by actuating the APL chamber situated between the two coil springs. It is worth mentioning that Mode III (Contraction) requires an additional constraining shell to suppress radial expansion, thereby amplifying inward radial contraction.

2. **Experimental calibration of the air pressure versus deformation under varying chamber widths of APL**

Focusing on the APL width parameter in the 10-30 mm range with 5 mm increments, we systematically investigated how pneumatic pressure influences BioflexBot’s final deformation, with results summarized in Fig. 2B. Comparing structural deformation behaviors across different APL widths revealed significant variations in pressure response.

1) For the APL with 10 mm width, the BioflexBot exhibited continuous radial expansion (expansion rate $\delta D/\delta P\approx0.12$ mm/kPa) within the 0-65 kPa pressure. However, when the pressure exceeded 65 kPa, the expansion rate decreased markedly, approaching zero (the purple line of Fig. 2B).
2) When the APL’s width was 30 mm, BioflexBot experienced a motion mode transition at 15 kPa, shifting from radial expansion to tangential elongation. The deformation process is shown in fig. S3.

This variation arises from the imbalance between extension forces of APL and restoring forces of coil spring: wider chamber exhibits dominant extension forces, directly resulting in the extension of BioflexBot. In contrast, narrower chamber demonstrates insufficient extending capacity, causing their deformation governed primarily by interlayer compressive forces during expansion. The intrinsic non-extensible character of the chambers limits their expansion, constraining the BioflexBot’s ultimate radial expansion diameter.

3. **Experimental investigation of controllable force manipulation in F-pinching mode**

To verify the controllability of force during the F-pinching process, we conducted two sets of experiments.

1) Simulated traditional acupuncture procedures: We preliminarily validate the BioflexBot’s suitability for medical applications following the protocol outlined in fig. S7. Initial F-pinch: Inflate the APU to stably pinch the tip of acupuncture needle (diameter: 1.1 mm). Insertion: Gradually increase pressure to enhance F-pinching force against slippage while robotically inserting the needle perpendicularly into a human tissue phantom. Extraction: Maintain pressure while extracting the needle.

2) Chemical liquid transfer: To further evaluate the precision manipulation, we conducted experiments illustrated in the fig. S8. Sampling: Inflate APU to compress the pipette tip for air expulsion, then release pressure to draw up the target solution by negative pressure. Transfer: Maintain pressure to prevent the pipette from slipping during lifting using BioflexBot. Dispensing: Control liquid release by either ramping pressure for dropwise dispensing or step-pressure actuation for continuous liquid ejection.

These experiments preliminarily validate the BioflexBot’s capacity to precisely regulate force during F-pinching, fulfilling the critical requirements of precision-demanding applications such as medical procedures and chemical laboratory, etc.

4. **Relationship between inflated air volume and rotation angle**

To quantify the relationship between the gripper’s rotation angle and the input air volume into APU, this study simplifies the gripper, due to the constraint of the coiled spring, into a standard Archimedean spiral model (fig. S9A). For an arbitrary point “p” on this spiral, its radial distance to the center $r$, which represents the instantaneous radius of the spiral at this point, is described as:

$$\begin{aligned} r=r_{0}+k\varphi,\#\left( 1 \right) \end{aligned}$$

where $r_{0}$ is the initial radius of the spiral, $k$ is a constant determining the tightness of the spiral, and the angle $\varphi$ represents the rotation angle of point “p” relative to the start point of the coil and calculated by $\varphi=2n\pi+\theta$. Here, $\theta\in[0, 2\pi]$ is the angle between the line connecting the point “p” to the center and the line from the starting point to the center, $n$ is the number of complete turns the point has made relative to the starting point ($n\in N$).

From the arc length integral formula, the arc length from the starting point to any point “p” on the gripper can be expressed as follows:

$$\begin{aligned} s=\int_{0}^{\varphi} \sqrt[2]{r^{2}+\left( \frac{dr}{d\varphi} \right)^{2}}d\varphi=\int_{0}^{\varphi} \sqrt[2]{{(r_{0}+k\varphi)}^{2}+k^{2}}d\varphi.\#\left( 2 \right) \end{aligned}$$

To determine the coil parameter $k$ and the final angle $\varphi_{end}$ at the gripper’s tip, the outer radius $R_{outer}$ of the gripper must first be established. Experimental observations show that during rotation, the inner radius $c$ of the gripper approximately equals the radius of the grasped object. When the object is grasped, tight interlayer contact of the robot causes $R_{outer}$ to increase. However, due to the influence of membrane bending and wrinkling, the interlayer thickness is unevenly distributed, making it difficult to predict $R_{outer}$ directly. To simplify the model, we obtained an empirical relationship about the gripper’s outer radius $R_{outer}$ through experimental fitting: $R_{outer}=r_{0}+t$, where $t$ is a constant coefficient determined experimentally. In this study, the values of $t$ were found to be 6, 9, and 14 for grasped objects with radii of 10 mm, 15 mm, and 20 mm, respectively.

Based on Equation (1) and (2), we can calculate the arc length of the gripper’s endpoint $s_{ep}$, by substituting the outer radius of the gripper $R_{outer}$ as $r$ and the angle $\varphi_{ep}$ as $\varphi$. Since we know that the arc length of the gripper’s endpoint equals to the total length of the gripper $L$, which is a constant value, we can get the equation $s_{ep}=L$. Then through numerical solution, the angle $\varphi_{ep}$ of the gripper’s endpoint, and the coil parameter $k$ can be obtained for target objects of different diameters.

Assuming the elongated length of the gripper during rotation is $L_{e}$ (fig. S9B), and the angle rotation angle $\gamma$ can be obtained using equation bellow:

$$\begin{aligned} L_{e}=L-\int_{0}^{\varphi_{ep}-\gamma} \sqrt[2]{\left( r_{0}+k\varphi\right)^{2}+k^{2}}d\varphi.\#\left( 3 \right) \end{aligned}$$

To establish the relationship between the inflation volume $V$ and the rotation angle $\gamma$, the relationship between the inflation volume $V$ and the gripper’s elongation $L$ length must be further clarified. Due to the compressibility of air, the actual volume change of air inside the gripper is relatively complex. Therefore, we employed a piston cylinder platform to precisely control the actual volume of air injected into the gripper, which is considered as the inflation volume $V$. The relationship between the volume $V$ and the gripper’s elongation length $L_{e}$ was determined through experimental calibration, with the results shown in Fig. 3B. Based on the experimental data, the numerical relationship can be established via polynomial fitting, expressed as follows:

$$\begin{aligned} L_{e}=aV+{bV}^{2}-c,\#\left( 4 \right) \end{aligned}$$

where $a=1.867$, $b=0.0076$, and $c=12.516$. By solving equations (3) and (4), the rotation angle $\varphi$ of the object driven by the gripper under different input inflation volumes $V$ can be numerically obtained.

5. **FP-grasping mode for gripping various objects**

We evaluated the effectiveness of the robot in objects hooking, with results shown in Fig. 5C, fig. S13, and Movie 3. The operational sequence of the FP-grasping is as follows:

1) Inflate the APU to extend BioflexBot while maintaining a bent tip.

2) Make contact between the bent tip and target object.

3) Partially deflate APU to induce contraction and helical winding to catch the object tightly.

These demonstrations confirm BioflexBot’s capability to hook both annular objects (e.g., toolbox handles, headphone arcs) and protruding body (e.g., goggles with frame protrusion, slotted-top containers).

Supplementary Figure 1. Heating press process to fabricate the structure of the robot.

Supplementary Figure 2. Experimental results of the pressure-induced deformation of BioflexBot with and without a constraining shell. Deformation process (A) without a constraining shell and (B) with a constraining shell. (C) Experimental relationship between the minimum inner gap and the air pressure.

Supplementary Figure 3. Experimental comparison of the pressure-induced deformation by varying chamber widths of APL. Deformation of the robot with a chamber width of (A)10 mm and (B) 30 mm.

Supplementary Figure 4. Parametric schematic of BioflexBot's grasping behavior in expansion mode. (A) When the central angle $\boldsymbol{\gamma<\pi}$, schematic illustration of the geometric interaction between the grasped object and the robot. (B) When $\boldsymbol{\gamma}$ > π, the object is partially enveloped but not fully enclosed, showing the geometric relationship with the robot. (C) The object is completely enclosed by the robot.

Supplementary Figure 5. The force analysis of F-pinch grasping. (A) Definition of the diameters of conical frustum objects to be grasped. (B) Force analysis under different diameters of the object.

Supplementary Figure 6. Demonstration of the F-pinch in standard objects holding. Objects include (A) a pen and (B) a straw.

Supplementary Figure 7. Acupuncture in a human tissue phantom leveraging the F-pinching function. (A) The Acupuncture needle and its diameter. (B) Demonstration of needle insertion. (C) Retrieval of the robot while leaving the needle successfully inserted in the phantom. (D) Successfully extraction of the needle from the phantom by the robot.

Supplementary Figure 8. Experimental process of liquid transfer leveraging the F-pinching. (A) Liquid sampling achieved by fully compressing the pipette bulb. (B) Dropwise and continuous liquid extrusion achieved through intermittent and sustained compression of the pipette bulb respectively.

Supplementary Figure 9. Parametric schematic of the relationship between robot extension length $\boldsymbol{L}_{\boldsymbol{E}}$ and innermost rotation angle $\boldsymbol{\gamma}$ in extension mode. (A) Schematic of the robot’s cross-sectional geometry in the extended state, based on idealized assumptions for modeling. (B) Geometric illustration of the coupling mechanism between axial extension length and the rotational angle.

Supplementary Figure 10. Demonstration of the hooking function for gripping various objects. Objects include (A) an over-ear headphone and (B) a box to store gloves.

Supplementary Figure 11. Experimental setup and test models for dynamic gripping force measurements.

Supplementary Figure 12. Results of the dynamic gripping force measurement. Force data recorded at different actuation pressures during grasping and release, obtained from the eight embedded sensors in (A) a cylinder mode and (B) a cube mode.

Supplementary Figure 13. Demonstration of the FP-grasping function for gripping various objects. The objects include (A) a tomato, (B) a fascia ball, (C) a paper box, (D) a pair of bananas, and (5) a 3D-printed vase.

Supplementary Figure 14. Demonstration of the large-scale grasping function for gripping various objects. Objects include (A) a test tube rack, (B) a transparent adhesive tape roll, and (C) a 3D-printed pen holder.

Supplementary Figure 15. Demonstration of the BioflexBot’s capability to grasp objects in complex channel environments. Channels include (A) curved channels and (B) angular channels.

Supplementary Figure 16. Demonstration of multiple chopsticks grasping and sequential transportation. (A) Grasping five pairs of chopsticks by inflating the APU, followed by sequential release via APU deflation. (B) Selective release positions and sequence for different chopsticks.

Supplementary Table 1. Comparison of existing soft end-effectors with our gripper

| **Reference No.** | **Control Method** | **Number of Control Input** | **Gripper Size**  (mm) | **Graspable Size**  (mm) | **Graspable Size Range** |
| --- | --- | --- | --- | --- | --- |
| 21 | Motor | 1 | 100 | 7-54 | 7.71 |
| 22 | Pneumatic | 1 | 120 | 36-85 | 2.36 |
| 23 | Pneumatic | 1 | 70 | 2-54 | 27 |
| 24 | Motor | 1 | 235 | 40-120 | 3 |
| 25 | SMA | 1 | 150 | 37-70 | 1.89 |
| 26 | Pneumatic | 2 | 90 | 25-90 | 3.6 |
| 27 | Magnetic | 2 | 40 | 1-48 | 48 |
| 28 | Pneumatic | 2 | 113 | 40-120 | 3 |
| 29 | Cable | 2 | 250 | 5.6-125 | 22.32 |
| 30 | Pneumatic &Jamming  (Universal gripper) | 2 | 20 | 2-20 | 10 |
| 31 | Cable | 2 | 200 | 1-180 | 180 |
| 32 | Pneumatic | 6 | 115 | 0.4-140 | 350 |
| 33 | Pneumatic & Magnetic | 4 | 160 | 20-60 | 3 |
| 34 | Cable | 4 | 115 | 3-160 | 53.33 |
| 35 | Pneumatic | 3 | 74 | 20-70 | 3.5 |
| 36 | Pneumatic | 4 | 210 | 10-200 | 20 |
| 37 | Pneumatic | 4 | 200 | 10-190 | 19 |
| 38 | SMA & Motor & Microwedge | 3 | 222 | 2-260 | 130 |
| 39 | Cable | 3 | 225 | 10-100 | 10 |
| 40 | Cable & Pneumatic & Jamming | 6 | 152 | 1-250 | 250 |
| 41 | Pneumatic | 9 | 200 | 20-200 | 10 |
| 42 | Pneumatic | 8 | 204 | 1.5-52 | 34.67 |
| 43 | Pneumatic & Cable | 7 | 200 | 67-67 | 1 |
| 44 | Cable & Pneumatic & Jamming | 9 | 100 | 10-100 | 10 |
| **Ours** | Pneumatic | **2** | **40** | **0.09-210** | **2333.33** |

Supplementary Table 2. Bill of Materials

| **Materials** | **Sources** | **Specifications** | **Cost** |
| --- | --- | --- | --- |
| Single TPU coated fabric for APU | Jiaxing Yingcheng Textile Co., Ltd. | Length: 230 mm  Width: 50mm  Thickness: 0.2 mm | $0.5 |
| Double TPU coated fabric for APU | Jiaxing Yingcheng Textile Co., Ltd. | Length: 230 mm  Width: 50 mm  Thickness: 0.2 mm | $0.5 |
| Single TPU coated fabric for APL | Jiaxing Yingcheng Textile Co., Ltd. | Length: 230 mm  Width: 35 mm  Thickness: 0.2 mm | $0.5 |
| Coiled spring | Shenzhen Dongji Spring Co., Ltd. | Length: 240 mm  Width: 4 mm  Thickness: 0.2mm  Quantity: 2 | $1 |
| PU tube | Yueqing Hebai Pneumatic Co., Ltd. | Length: 20000 mm  Inner diameter: 1 mm  Outer diameter: 2 mm | $1 |
| Glue | Shenzhen Jinsibang Adhesive Co., Ltd. | Ergo 5800 | $0.5 |
| Constrain shell | 3D-Printed in Laboratory | Inner diameter: 35 mm  Outer diameter: 40 mm | $1 |
| In Total: $5 | | | |

Supplementary Table 3. Dexterous Hand can be Purchased

| **Name** | **DoF** | **Company** | **Costs** | **Actuation** |
| --- | --- | --- | --- | --- |
| FlexHand V1 | 6 | SoftSync Robotics Limited | $500 | Tendon driven |
| DexHand021 | 19 | DexRobot | $10,000 | Tendon driven |
| Sharpa Wave | 22 | Sharpa | $50,000 | Motor direct driven |
| Revo 2 | 11 | BrainCo | $5,000 | Motor direct driven |
| Linker Hand L20 | 20 | Linkerbot Technology Co., Ltd. | $7,000 | Linkage driven |
| FDH-6 | 6 | Fourier | $700 | Linkage driven |

Movie S1.

**Human-like grasping through bio-functional mimicry (F-pinch).** This video demonstrates two capabilities of the BioflexBot in the f-pinch function. (1) Ultrafine grasping capability enables stable and reliable grasping across scales, from a human hair (approximately 0.09 mm in diameter) to a standard-sized pen. (2) the robot precisely regulates pinching force to perform delicate tasks: 1) insertion and extraction of a needle into/from a tissue phantom, simulating acupuncture procedures; and 2) precise and controllable transfer of liquids, showcasing potential applications in biomedical sample handling.

Movie S2.

**Human-like grasping through bio-functional mimicry (Rotate).** This video presents two applications of the rotate function. First, it demonstrates continuous unscrewing of a cylindrical bottle cap, achieving a rotation of 690° in a single motion, which far exceeding typical human hand’s capability; Second, through fine control of rotational angle, the robot performs precise orientation-based assembly by inserting a square peg into its corresponding hole, validating its high-precision angular regulation.

Movie S3.

**Human-like grasping through bio-functional mimicry (Hooke & FP-grasp).** This video illustrates the operational performance of two additional functions when interacting with various objects. Utilizing the hooking function, BioflexBot successfully and stably hooks objects with engageable structures, such as a ring handle and a toolbox. Then, using the FP-grasping function, it achieved reliable gripping of objects with diverse shapes, including spheres, complex curved surfaces, and prisms.

Movie S4.

**An upper limb with adaptive growing capabilities (Large-scale grasping & Long-distance operation).** This video demonstrates the BioflexBot’s ability to grasp objects significantly larger than itself, with internal cavity sizes ranging from 1.2 to 5 times its own dimensions and diverse cavity shapes such as rectangular, circular, and hexagonal profiles. Additionally, with its base fixed, the BioflexBot alone can perform long-distance pushing and pulling of a drawer through the coordinated extension and contraction, demonstrating its capability for remote spatial operations.

Movie S5.

**An upper limb with adaptive growing capabilities (Confined space delivery). This video shows BioflexBot navigating complex confined environments.** First, it successfully traverses a straight, narrow channel to grasp and deliver a pair of scissors. Next, it adapts its shape to smoothly pass through the channels with intricate geometry, completing the task of delivering the target object.

Movie S6.

**An upper limb with adaptive growing capabilities (Multi-object sequential transportation).** This video highlights the robot’s capability to realize multi-object sequential transportation through the coordinated motion modes. The robot first sequentially grasps various objects, including a perfume bottle and several pens, and transports them into different containers. Subsequently, it completes the on-demand transportation of multiple pairs of chopsticks based on the same principles.

Movie S7.

**Tool-Hand integration (Aeroengine blades inspection).** This video demonstrates the use of two BioflexBot connected in series to inspect aeroengine blade arrays, highlighting their integrated tool-hand functionality. The system not only successfully completed the inspection task for five points in regular positions but also effectively reached and inspected difficult-to-access points on the trailing edge of blade, validating its accessibility within deep, complex, and confined spaces.

Movie S8.

**Tool-Hand integration (Hand for humanoid robot).** This video showcases the performance of the robot when integrated as a dexterous hand at the end of a humanoid robot. It performs stable grasping, obstacle-avoiding grasping, and compliant operations during safe human-robot interaction tasks, illustrating both precision and adaptability.

Movie S9.

**Integrated multifunction.** This video captures BioflexBot operating in a complex, multi-task automated chemical reaction experiment. Beginning with a vision-based system locating, it proceeds with precise grasping and targeted placement of reaction vessels. Finally, through fine pressure control, it achieves the transfer of chemical reagents, successfully triggering a colorimetric reaction that visually represents the chemical process.
